# Muscle strength does not explain standing ability in children with bilateral spastic cerebral palsy: a cross sectional descriptive study

**DOI:** 10.1186/s12883-015-0441-y

**Published:** 2015-10-08

**Authors:** Cecilia Lidbeck, Kristina Tedroff, Åsa Bartonek

**Affiliations:** Department of Women’s and Children’s Health, Karolinska Institutet, Stockholm, Sweden

**Keywords:** Asymmetry, Cerebral palsy, Child, Isometric muscle strength, Posture

## Abstract

**Background:**

In bilateral cerebral palsy (CP) muscle strength is considered important for development of gross motor functions, but its influence on standing ability has not been explored. Our aims were to examine muscle strength with respect to the ability to stand with (SwS) or without (SwoS) hand support, asymmetrical weight bearing (WB), and whether the ability to produce strength was influenced by different seated conditions.

**Methods:**

In this cross sectional descriptive study standing posture was recorded with 3D motion analysis, and muscle strength was measured with a hand-held dynamometer, in 25 children with bilateral CP, GMFCS levels II-III, SwS (*n* = 14, median age 11.4 years), or SwoS, (*n* = 11, median age 11.4 years). Strength measurements were taken in the hip flexors, knee extensors, dorsiflexors and plantarflexors, in two seated conditions; a chair with arm- and backrests, and a stool.

**Results:**

Compared to SwoS, children SwS stood with a more flexed posture, but presented with equal strength in the hip flexors, dorsiflexors and plantarflexors, and with somewhat more strength in the knee extensors. Despite asymmetric WB during standing, both limbs were equally strong in the two groups. No differences in strength were measured between the two seated conditions.

**Conclusions:**

Despite challenges measuring muscle strength in CP, the lower limb muscle strength cannot be considered an explanatory factor for variations in standing in this group of children with bilateral CP. The findings rather strengthen our hypothesis that deficits in the sensory systems could be as determinant for standing as muscle weakness in children with bilateral spastic CP.

## Background

Cerebral palsy (CP) is the most common childhood motor impairment in developed countries and describes a group of permanent disorders of the development of movement and posture. The motor disorders are often accompanied by disturbances of sensation, perception and cognition [1]. In bilateral CP muscle strength is considered important for development of gross motor functions, but its influence on standing ability has not been explored. Muscle weakness is recognized as a primary symptom in children with CP and children with spastic CP are substantially weaker compared to typically developing (TD) children in the lower limb muscle groups, most prominently in the ankle and the hip muscles [2, 3]. When muscle strength in CP was analyzed from a gross motor function classification system (GMFCS) perspective, the children with better gross motor function, GMFCS level I, proved to be the strongest [2, 4, 5]. Muscle strength has been considered important for both gross motor function development and walking ability in children with bilateral CP [2, 6]. Children with bilateral CP frequently have difficulties with upright standing and some may require hand-support. It is unclear however, if the development of standing ability is dependent on muscle strength or if there are other factors affecting the ability to attain standing in CP.

Muscle strength is clearly important for achieving gross motor functions. Since children with CP are weak, resistance training to achieve functional benefits has become a commonly recommended intervention. Disappointingly, functional gains following resistance training in children with CP have been hard to demonstrate even though increased muscle strength was detected [7, 8]. In children with CP the motor disorders are often accompanied by disturbances of sensation and perception [1]. Investigation on how deficits in the sensory systems affect the motor disorder in CP are reported in few studies only. The discrepancy between the children’s gross motor function score and how they use their motor function in everyday life, has been hypothesized to be associated with perceptual disorders [9]. In another study by Ferrari et al., an impaired motor control strategy with ineffective anticipatory postural adjustments during a seated reaching task in children with bilateral CP, was explained by disturbances in the perceptual system [10]. Difficulties in producing antigravity reactions during standing have been proposed to relate to perceptual problems [11]. Furthermore, Damiano et al. found that deficits in proprioception with impaired detection of joint position in the lower limbs were linked to postural instability in standing in children with mild bilateral CP [12]. The above mentioned findings indicate that disorders in the sensory and perceptual systems affect gross motor function in CP even if they are not fully understood.

Compared to children with typical development (TD), children with bilateral CP often present with a flexed standing posture. We have previously found that children stood with more flexion than their potential passive joint angle, showing that they had less restricted knee extension in a non-weight-bearing supine position than in a weight bearing (WB) standing condition [13]. According to the 2007 CP definition, some children categorized with primarily bilateral involvement may have considerable asymmetry across sides [1]. In our previous study, the assessment of standing posture in children with bilateral CP revealed a more apparent asymmetrical alignment in the children who stood independently compared to those who needed support [13]. When investigating muscle strength between the more and the less involved limb results indicated that there were no differences in muscle strength between the limbs in bilateral CP [3].

When measuring isometric muscle strength, information about the ability to produce force at a specific, position-dependent muscle length is provided. Measuring muscle strength in children, and in particular in children with brain lesions has been reported to be demanding and deserves careful standardization of measuring positions and data comparisons [14–16]. The reliability of measuring muscle strength in children with CP is challenging due to poor selective motor control, co-contraction, and the restriction of joint range of motion [3, 14, 17]. Still, measuring muscle strength with a hand-held dynamometer (HHD) in children with CP has been reported as reliable in the lower-extremities and is a commonly used method in clinical practice [15, 18, 19]. Although, the value of measuring muscle strength in one position and then relate results to gross motor functions, where the muscles are working in other lengths, have been questioned [14].

To our knowledge, there is no information about the role of muscle strength for standing ability in children with CP. The aims were therefore: (i) to explore the lower limb muscle strength in children with various standing abilities, both those requiring assistive device and those who stood unsupported; (ii) to investigate muscle strength with respect to asymmetric WB during standing; and (iii) whether children’s ability to produce strength was influenced by different seated conditions. Based on the theory that in CP, the motor disorders are accompanied by disturbances of sensation and perception [9–12], our hypothesis was that in children with bilateral CP, sensory disorders would be as determinant as muscle weakness for standing function in CP.

## Methods

### Participants

In this cross sectional descriptive study standing posture and muscle strength were investigated in children and adolescents with CP. Participants were consecutively recruited through the neuropaediatric department of Karolinska University Hospital in Stockholm, Sweden, between January 2012 and September 2013. The inclusion criteria were; bilateral CP, 7–17 years of age, GMFCS level II–III, the ability to maintain standing with (SwS) or without (SwoS) hand support for at least 30 s, and the ability to follow verbal instructions. Exclusion criteria were; presence of dystonia, botulinum toxin injections or soft tissue surgery within the past six months, or skeletal surgery within the past year. Informed consent was obtained, verbally from the children and written from the parents. Approval was obtained by the Regional Ethical Review Board in Stockholm, Sweden.

### Physical examination

All children underwent a physical examination that included measurements of joint range of motion (ROM) [20]. If passive ROM was less than the neutral position of a joint, the joint was considered to have a contracture. Spasticity in the lower limbs was also assessed and was documented as either present or absent (Table 1) [21].

**Table 1:** Characteristics of children with CP, standing with (SwS) and without (SwoS) hand support

	SwoS (*n* = 11)	SwS (*n* = 14)
Age, median (range), years	11.4 (7.7–15.9)	11.4 (7.7–17.2)
Weight, median (range), kg	45.0 (30.7–99.7)	37.3 (18.5–65.2)
Height, median (range), cm	150.0 (124.0–166.0)	141.0 (110.0–170.0)
Female/male	7/7	5/6
GMFCS Level	II: 9, III: 2	II: 1, III: 13
Joint contractures, uni or bilateral^a^		
Hip flexion contracture	4	8
Knee flexion contracture	4	11
Plantarflexion contracture	5	5
Spasticity, uni or bilateral^a^		
Hip flexors	1	2/13
Knee flexors	10/10	14
Plantarflexors	11	14
Orthopaedic surgery^a^		
Femoral osteotomy	3	3
Adductor tenotomy	0	1
Hamstrings lengthening	0	1
Calf (Strayer, Achilles tenotomy)	6	4

^a^nr of children

### Motion analysis

The standing posture was recorded with a three-dimensional (3D), eight camera motion analysis system (Vicon MX40®^,^ Oxford, UK) using a full-body biomechanical model (Plug-In-Gait, Vicon®) with retro-reflective markers, during 30 s while standing on two force plates (Kistler®, Winterthur, Switzerland). The children who needed hand support to achieve and maintain standing stood in front of a height-adjustable frame and held the handrail with a slightly flexed elbow position. All children were tested barefoot. The more WB limbs were determined from force plate data. The mean and standard deviation (SD) sagittal plane trunk and pelvis segment, and hip, knee and ankle joints angles from each child’s standing trial were used to describe standing posture.

### Muscle strength measurements

A hand-held dynamometer (Chatillon®, Greensboro, NC, USA) was used to quantify isometric muscle strength. Four muscle groups were tested bilaterally: hip flexors, knee extensors, ankle dorsiflexors, and ankle plantarflexors. Lever arms were measured from the greater trochanter, lateral knee joint, and lateral malleolus to the dynamometer’s perpendicular placement. The strength measurements were carried out in randomized order in two seated conditions; a chair with arm- and backrests (chair), and a stool after removing arm- and backrests (stool).

Children were instructed to place their hands on their laps during measurements, and not to lean against the armrest or the backrest. The hip flexors and knee extensors were tested in 90° hip flexion and 90° knee flexion as previously described by Eek et al. (Table 2) [22]. To avoid possible restrictions from tight hamstring and/or gastrocnemius muscles the dorsiflexors and plantarflexors were tested in 90° hip flexion and 30° knee flexion. The ankle was passively placed in a neutral position. If the range of motion in the ankle was limited, the HHD was placed as close to neutral position as possible [3].

**Table 2:** Test positions for isometric muscle strength measurements with hand-held dynamometer

	Muscle	Position in sitting	Lever arm from
	Hip flexors	Hip and knee flexed 90°	Greater trochanter
	Knee extensors	Hip and knee flexed 90°	Lateral knee joint
	Ankle dorsiflexors	Knee flexed 30°	Lateral malleolus
		Ankle neutral	
	Ankle plantarflexors	Knee flexed 30°	Lateral malleolus
		Ankle neutral	

The “make test technique” [14] was used by verbally encouraging the children to press as hard as possible against the dynamometer to build up strength for 4–5 s. Between the trials there was a break of approximately 20 s. To ensure understanding children first completed a familiarization trial. Three trials for each muscle group were conducted in each seated condition. The same examiner (CL) performed all measurements while another controlled for standardization of the testing positions. The force value derived from the dynamometer was multiplied with the lever arm to express strength as torque, which was in turn normalized to body weight. Muscle strength data was analyzed with respect to children’s standing ability, more-WB and less-WB limb and the seated conditions.

### Statistical analysis

Non-parametric statistical analyses were carried out using the commercially available software SPSS v.21 Chicago, IL, USA, at a significance level of p ≤ 0.05. A Mann–Whitney *U* test was used for group comparisons between SwS and SwoS. A Wilcoxon’s Signed- Rank Test was used to compare muscle strength between the more-WB and less-WB limbs and chair or stool conditions. A Chi-Square test was used to compare incidence of joint contractures, spasticity, and performed orthopedic surgery in the lower limbs between the two groups.

## Results

### Participants

Of the 27 children who were enrolled in the study, two did not complete the examinations; thus 25 children (12 females and 13 males), median age 11.4, (range 7.7-17.2) years participated. According to standing ability 14 children required hand support during standing (SwS), and 11 were able to stand without hand support (SwoS) (Table 1).

No differences were found between children in the SwS-group or SwoS-group in age, weight, height, gender, presence of spasticity or amount of orthopedic surgery. Knee flexion contractures were significantly more frequent in children SwS vs SwoS (*p* = 0.049). The number of contractures were equally distributed in the hip (*p* = 0.428) and the ankle (*p* = 0.697) (Table 1). Among the children with contractures, in SwS and SwoS respectively, the passive ROM median (range) in hip extension was −5° (−5-(−15)° and −12.5° (−10-(−20)°, in knee extension: −15° (−10-(−25)° and −15 (−5-(−25)°, and in the ankle dorsiflexion: −5° (−5-(−20)° and −5° (−5-(−25)°. Minus is indicating presence of a flexion contracture.

### Standing posture

**Fig. 1:**
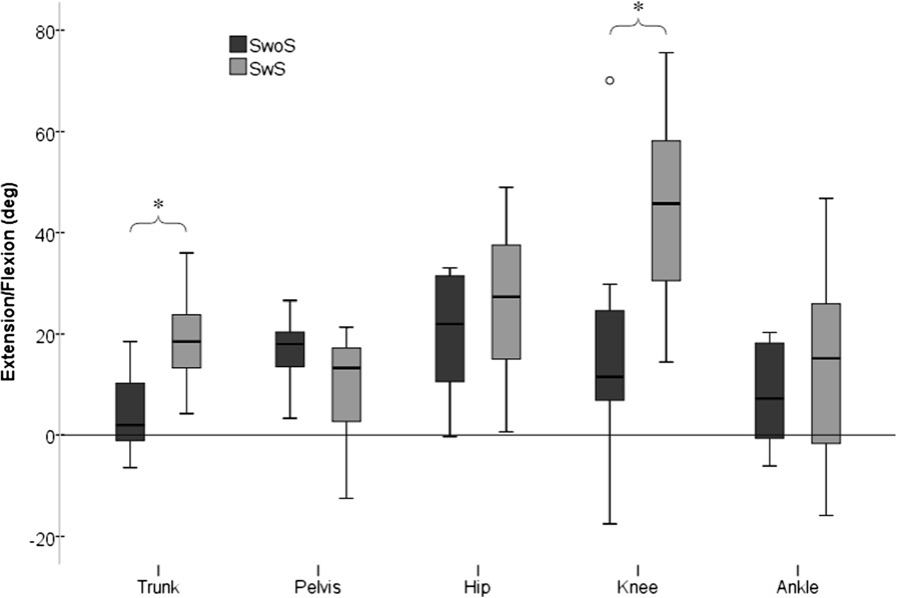
Standing posture in children with CP, standing with and without hand support. Illustration of sagittal plane segment and joint angles (degrees) during standing, measured with 3D motion analysis, in the more weight bearing limb, in children with bilateral CP, standing with (SwS) and without (SwoS) hand support. Data is presented as median and 25th and 75th percentiles, min and max values, °represent outliers. Negative values (−) indicate extended trunk, backward tilted pelvis, hip extension, knee hyperextension, and ankle plantarflexion. *Indicate significant difference between SwS and SwoS, calculated with a Mann–Whitney *U* test (p ≤ 0.05)

On a group level, the 3-D motion analysis showed that all children stood with forward leaning trunks, anteriorly tilted pelvis, flexed hips and knees, and with the ankle in dorsiflexion. There was large heterogeneity within the SwoS and SwS groups. Compared to children in SwoS, the children in SwS stood with significantly more forward trunk lean, 19.2 versus 4.4° (p < 0.001), and with more knee flexion, 44.4 versus 15.8° (*p* = 0.001). The joint angles in SwS were also somewhat greater in the hip and ankle, though not significantly (Fig. 1).

**Fig. 2:**
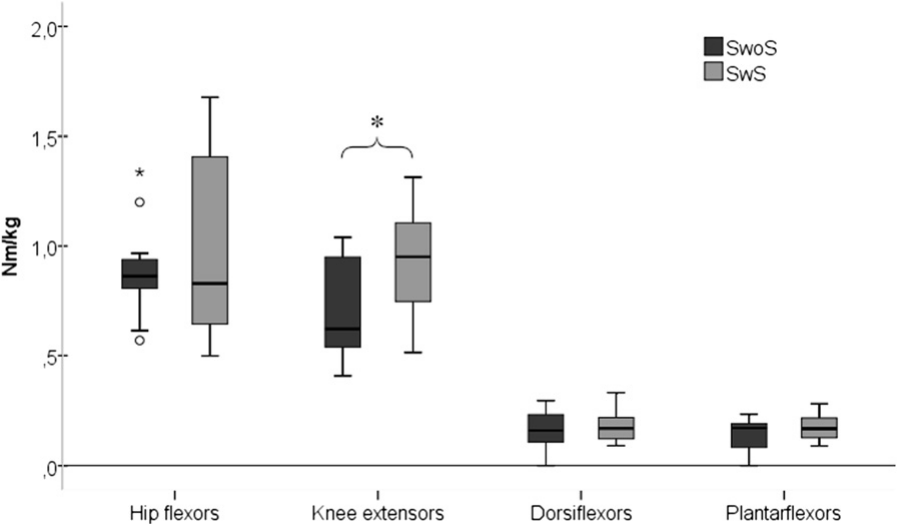
Muscle strength in children with CP, standing with and without hand support. Illustration of muscle strength (Nm/kg) when sitting in a Chair, obtained from the more weight-bearing limb, in children with bilateral CP, standing with (SwS) and without (SwoS) hand support. Data is presented as median and 25th and 75th percentiles, min and max values, °represent outliers, and *represent extremes. *Indicate significant difference between SwS and SwoS, calculated with a Mann–Whitney *U* test (p ≤ 0.05)

Differences in weight distribution between the limbs were identified in all children. The more-WB limb was generally more extended in both SwS and SwoS, although there were large intragroup variations. For both children SwoS and SwS the hip was significantly less flexed in the more-WB limb, 19.9° vs 24.8° (*p* = 0.026) and 25.9° vs 34.4° (*p* = 0.006) respectively. In addition the SwS group had a more extended knee in the more-WB limb, 44.4° vs 52.6° (*p* = 0.041). No differences were observed in the ankle.

### Muscle strength

Seventeen of the 25 children included in the study lost attention during testing and completed one trial only in each muscle group in the second position. The first trial from each muscle group was therefore, used for analysis.

There were no differences in muscle strength between the limbs with respect to asymmetrical WB in the hip flexors, knee extensors, or the plantarflexors in either the SwS-group or the SwoS-group. Only the dorsiflexors showed significantly higher strength in the more-WB limb in children SwS (Table 3).

**Table 3:** Muscle strength in children with CP standing with (SwS) and without (SwoS) hand support

	More WB-limb		Less WB-limb		
	Median (Range) Nm/kg	n	Median (Range) Nm/kg	n	*P*
SwoS (*n* = 11)					
Hip flexors	0.86 (0.57–1.34)	11	0.81 (0.52–1.37)	11	0.790
Knee extensors	0.62 (0.41–1.04)	11	0.82 (0.39–1.24)	11	0.328
Dorsiflexors	0.16 (0.00–0.30)	10	0.15 (0.00–0.30)	11	0.441
Plantarflexors	0.17 (0.00–0.23)	9	0.15 (0.00–0.33)	9	0.575
SwS (*n* = 14)					
Hip flexors	0.83 (0.50–1.68)	14	1.04 (0.53–1.79)	14	0.331
Knee extensors	0.95 (0.51–1.31)	14	0.87 (0.44–1.56)	14	0.177
Dorsiflexors	0.17 (0.09–0.33)	13	0.12 (0.08–0.24)	13	**0.034**
Plantarflexors	0.17 (0.09–0.28)	13	0.17 (0.08–0.27)	13	1.000

P-values refer to comparisons between the limbs, significant difference is bolded (*p* ≤ 0.05)

*n* = nr of children

Between the two groups in the more-WB limb, the knee extensor strength values were significantly higher in SwS than in SwoS (*p* = 0.038). No significant differences between SwS and SwoS were found in the hip flexors (*p* = 0.767), dorsiflexors (*p* = 0.976), or the plantarflexors (*p* = 0.431) (Fig. 2).

There was no statistical difference in muscle strength between the seated conditions in either SwS or SwoS.

## Discussion

Measuring isometric muscle strength in children with CP is associated with uncertainties not least because of their deficits in performing voluntary movements [23]. Despite difficulties involved with the measurements, strength does not seem to be a determining factor in whether the children with CP need support during standing; only minor differences were found in lower limb muscle strength between children standing with or without support. The need for support, as well as a more apparent crouched standing posture in the same individuals, might lead us to believe that difficulties during standing originate from muscle weakness. But contrary to this assumption, the children in this study, were equally strong in their hip and ankle muscles, and the children who required support were somewhat stronger in the knee extensors. The strong knee muscles, however, might be a consequence of the more flexed knees during standing observed in the children SwS. On the other hand, these children stood not only with increased knee flexion, but with a more forward leaning trunk compared to the children in SwoS. As an effect of the flexed body position, the projection of the ground reaction force can be assumed as shifted anteriorly, reducing the internal knee extension joint moment and therefore decreasing the required effort of the knee muscles. Accordingly, the crouched posture during standing in this group of children may not have required much stronger knee muscles than in those standing independently. Furthermore, calf muscle weakness causing instability in the ankle joint may contribute to the flexed knee position during standing. In our study group, the plantarflexors were equally strong in both groups and there were no differences in the amount of calf muscle surgery between the groups. Secondary musculoskeletal problems with decreased joint ROM is frequently observed in children with CP [1, 24]. In our study, hip, knee and ankle contractures were equally pronounced in both groups, whereas knee flexion contractures were more common in the SwS-group. The knee contractures were not believed to negatively influence the children’s ability to produce maximal knee extensor force, as the measurements were performed with a 90° flexed knee.

In a previous study we reported that children with need for support in standing, stood with less knee extension than was passively available in a non-weight bearing position [13]. This finding could be confirmed in the present study where the children stood with more flexed knees than necessary with respect to their passive ROMs. Not utilizing the full possible knee extension range may indicate difficulties in producing antigravity reactions during standing as a consequence of perceptual problems [11]. Furthermore, impaired proprioception in the lower limbs has been reported to be associated with postural instability in bilateral CP and could have contributed to children’s difficulties to extend their legs against gravity [12].

In bilateral CP, an uneven weight bearing is frequently observed during standing, giving an impression of asymmetric muscle strength. We found that the children’s more-WB limb was more extended both in the children in SwS or SwoS. We hypothesized that the support limb, i.e. the more WB limb was the strongest, but this could not be verified despite an asymmetrical loading on the limbs while standing. This is in accordance with Wiley et al. who found both limbs equally strong in children with bilateral CP [3].

Even though there are known uncertainties regarding strength measurements in CP, the strength values obtained in this study correspond to previous findings [25]. Dallmeijer et al. employed a method similar to ours and showed equivalent muscle strength values in the knee extensors, dorsiflexors, and plantarflexors in 25 adolescents with bilateral CP, GMFCS level II-III [25]. In accordance with earlier studies comparing muscle strength between GMFCS levels II and III, the children in our groups, SwS and SwoS, were equally strong in the hip flexors and knee extensors when measured in 90° knee flexion [2, 5]. However, when measured in 30° knee flexion, Thompson et al. reported children in GMFCS level III to be weaker in the knee extensors compared with children in level II [5]. This may be explained by the more severe motor disorder in GMFCS level III with difficulties in performing voluntary movements with an almost extended knee. Contrary to the findings of Eek et al. who reported stronger dorsiflexors and plantarflexors in children in GMFCS level II compared to III [2], we found these muscle groups to be equally strong in both groups. The testing positions used in our study were chosen in order to, to the best of our knowledge, reduce the impact of spasticity, tight muscles, and co-contraction [14]. For example the testing of the dorsiflexors in a sitting position, with a slightly flexed knee, avoiding constraint from tight muscles could have enhanced the children’s ability to produce a maximal voluntary contraction. The possibility to observe the foot during testing may also have supported the ability to selectively perform the dorsiflexion as well as to compensate for probable difficulties in proprioception [14, 26]. On the other hand in our study, there were three children who could not undertake strength measurements of the calf muscles due to poor selective motor control distally, in the plantarflexors. All three had a passive range of motion in the ankle to at least neutral position, hence it was not reduced mobility in the ankle that prevented children to perform the movement. Worth noting is that two of these children had the ability to stand without support and therefore, could be expected to have a milder motor disorder compared to children who required support for standing.

To examine whether the children’s ability to produce strength was influenced by various seated conditions, the measurements were conducted in a stable sitting position on a chair and in a more demanding sitting position on a stool. Contrary to Ferrari et al. we could not find any differences between the two conditions and speculate that children in our study were allowed to remain within their safe base of support at both measurements. In the above mentioned study, children’s limits of stability were provoked during a seated reaching task, thus our method might not have been challenging enough for the sensory-motor system to elicit possible perceptual impairments [9, 10].

A limitation of our study was the choice to use the first trial from each seated condition for the analyses. In order to compare muscle strength between groups, it has been recommended to use the highest value alternative an average of two trials for statistical analyses [27, 28]. However, most of the children had trouble to maintain attention during the testing, and it was demanding to accomplish three strength measurements per muscle group in the two seated conditions. Calculations on the maximum strength values from each muscle group were carried out with the same outcome and therefore, the choice to use data from the first trial does not seem to have affected the results. When using an HHD it is difficult to separate out forces caused by strength from spasticity or non-neural components as contractures. Since spasticity in the calf muscle could be expected to be easily elicited during the measurements as well as some children had contractures in the ankle, plantar flexor data from our study must be interpreted with caution. Our results though, were remarkably similar to the values from the Australian study, conducted on adolescents with CP [25].

## Conclusions

Despite the above discussed limitations and those associated with muscle strength measurement in children with CP in general, our results indicate that the children who required hand support compared to those who stood independently were not weaker in the lower limb muscles. Thus, lower limb muscle strength cannot be considered an explanatory factor for variations in standing in this group of children with bilateral CP. The findings rather strengthen our hypothesis that deficits in the sensory systems, such as proprioception and perception of gravity, could be as determinant for standing as muscle weakness in children with bilateral spastic CP. How disturbances in the sensory systems affect standing ability in children with bilateral CP will be further explored.
